# Ultrasound Assessment of Diaphragm Thickness and Thickening: Reference Values and Limits of Normality When in a Seated Position

**DOI:** 10.3389/fmed.2021.742703

**Published:** 2021-10-27

**Authors:** Alain Boussuges, Sarah Rives, Julie Finance, Guillaume Chaumet, Nicolas Vallée, Jean-Jacques Risso, Fabienne Brégeon

**Affiliations:** ^1^ERRSO, Institut de Recherche Biomédicale des Armées (IRBA), Toulon, France; ^2^Center for Cardiovascular and Nutrition Research (C2VN), Aix Marseille Université, INSERM, INRAE, Marseille, France; ^3^Service d'Explorations Fonctionnelles Respiratoires, CHU Nord, Assistance Publique des Hôpitaux de Marseille et Aix Marseille Univ, IRD, APHM, MEPHI, IHU-Méditerranée Infection, Marseille, France; ^4^ALTRA BIO SA, Lyon, France

**Keywords:** chest ultrasonography, hemidiaphragm, thickening fraction, thickening ratio, respiratory maneuvers

## Abstract

**Background:** Diagnosing diaphragm dysfunction in the absence of complete paralysis remains difficult. The aim of the present study was to assess the normal values of the thickness and the inspiratory thickening of both hemidiaphragms as measured by ultrasonography in healthy volunteers while in a seated position.

**Methods:** Healthy volunteers with a normal pulmonary function test were recruited. The diaphragmatic thickness was measured on both sides at the zone of apposition of the diaphragm to the rib cage during quiet breathing at end-expiration, end-inspiration, and after maximal inspiration. The thickening ratio, the thickening fraction, and the thickness at end-inspiration divided by the thickness at deep breathing were determined. The mean values and the lower and upper limits of normal were determined for men and women.

**Results:** 200 healthy volunteers (100 men and 100 women) were included in the study. The statistical analysis revealed that women had a thinner hemidiaphragm than men on both sides and at the various breathing times studied. The lower limit of normality of the diaphragm thickness measured at end-expiration was estimated to be 1.3 mm in men and 1.1 mm in women, on both sides. The thickening fraction did not differ significantly between men and women. In men, it ranged from 60 to 260% on the left side and from 57 to 200% on the right side. In women, it ranged from 58 to 264% on the left side and from 60 to 229% on the right side. The lower limits of normality of the thickening fraction were determined to be 40 and 39% in men and 39 and 48% in women for the right and left hemidiaphragms, respectively. The upper limit for normal of the mean of both sides of the ratio thickness at end-inspiration divided by the thickness at deep breathing was determined to be 0.78 in women and 0.79 in men.

**Conclusion:** The normal values of thickness and the indexes of diaphragmatic function should help clinicians with detecting diaphragm atrophy and dysfunction.

## Introduction

Measurement of the diaphragm thickness at the zone of apposition of the diaphragm to the rib cage has been used to detect diaphragm paralysis (1). It has been reported that a paralyzed diaphragm does not thicken significantly or become thinner upon deep inspiration compared with the thickness at end-expiration. A threshold of 20% is accepted by most authors for the diagnosis of hemidiaphragm paralysis (2). Diaphragm dysfunction in the absence of complete paralysis remains difficult to diagnose, however. Detection of such dysfunction is important because, although unilateral diaphragm weakness can remain asymptomatic in some patients, it can negatively impact the quality of life in some subjects, in particular those with underlying obesity or cardiorespiratory diseases. In this context, various clinical conditions such as orthopnea, coughing, chest pain, dyspnea on exertion, or sleep-disordered breathing can be observed (3, 4). Lastly, impairment of diaphragmatic function is a marker of disease severity in a number of neurological and muscular diseases such as amyotrophic lateral sclerosis (ALS), Duchenne muscular dystrophy (DMD), myotonic dystrophy, and myasthenia gravis (5).

A diagnostic approach of diaphragm dysfunction could be provided based on the lower limit value of normality of thickness and thickening. Several studies have determined the normal values of thickness measured at end-expiration and at end-inspiration in healthy volunteers in a supine position (6–10). To assess the quality of diaphragmatic function, the thickening ratio (TR) i.e., the thickness at end-inspiration divided by the thickness at end-expiration has been determined. The normal value for the TR has been estimated to be between 1.7 and 2 (6, 7, 9, 11). In some subjects, such as patients suffering from respiratory failure secondary to pulmonary or neuro-muscular diseases, the supine position is not well tolerated. Furthermore, it has been demonstrated that both the diaphragm thickness (12) and inspiratory thickening (13) are greater in sitting and standing positions than in the supine position.

By studying 45 healthy subjects, Brown et al. (13) determined that the normal percentage of thickening (the ratio of thickness at end-inspiration-thickness at end-expiration divided by thickness at end-expiration) was 65% when supine, 97% when seated and 174% when standing. Consequently, the lower limit of normal determined from studies performed in the supine position cannot be used when patients are placed in a seated position. Few authors have studied volunteers in semi-recumbent, recumbent, or sitting positions (13–15). Accurate normal values cannot be determined from these studies. Indeed, although it is recognized that gender and side have an impact on diaphragm thickness, in these previous studies the methods were not designed to determine normal values in men and women for both hemidiaphragms.

Lastly, the ratio between diaphragm thickness at the end of tidal volume and the diaphragm thickness at maximal inspiration (ΔTmax) has been proposed by Fantini et al. (16) to detect impairment in pulmonary function tests and as an indication for mechanical ventilation support in patients suffering from ALS. Indeed, the ΔTmax has been related to respiratory function tests (17). Although this ratio might be a relevant index of diaphragmatic function, to date, the normal values have been only estimated based on a small sample.

The present study was designed to determine the normal values of diaphragmatic thicknesses and the indexes of diaphragmatic function such as the thickening ratio and the ΔTmax based on a large population of healthy volunteers of both genders investigated while in a seated position.

## Materials and Methods

To recruit healthy volunteers, weekly medical consultations were conducted from January 2019 to January 2021. The research was conducted according to the Helsinki Declaration. The study protocol was approved by the Ethics Committee of Aix Marseille University (CPPRB 1, NoA01299-32). Written informed consent was obtained from all healthy volunteers. The volunteers were non-smokers and did not suffer from sarcopenia or morbid obesity. They were considered to be healthy if they did not have a history of cardio-respiratory disease or thoracic trauma and no clinical impairments at the time of the examination. Volunteers were sedentary or occasionally engaged in recreational sports activities, no athletes with a high level of physical training was included in the study. Furthermore, the pulmonary function test of the volunteers had to be normal. Pulmonary function was assessed with a spirometer (Ilmeter 1,304; Masterlab Jaeger, Wurzberg, Germany) according to the ERS/ATS standards (18). The criteria for classifying a pulmonary function test as normal were a slow vital capacity (SVC), forced vital capacity (FVC), and forced expiratory volume in 1 s (FEV 1) higher than the lower limit of normal of the reference population and a FEV1 to VC ratio greater than 0.7. Lastly, the diaphragmatic motion of both hemidiaphragms during quiet breathing and deep breathing was recorded in all of the volunteers using M-mode ultrasonography. Ultimately, to include the volunteers, the excursions had to be greater than the recently published lower limits of normality (19).

### Calculation of the Sample

Previous studies have reported that diaphragmatic thickness is greater in healthy men than in healthy women. To find differences between genders and appropriate normal values for men and women, the calculation of the sample was based on the results of Cardenas et al. (14) as this study used the same measurement method as ours. These authors reported a diaphragm thickness at the end of expiration of 1.9 ± 0.3 mm in men and 1.79 ± 0.3 mm in women. For a 0.05% alpha risk and an 80% power, we determined that at least 92 subjects would have to be included in each group (men and women). Since measurement of the diaphragm thickness can be difficult to perform on both sides in some volunteers, to increase the accuracy of the results, we aimed for a sample of 100 volunteers in both groups.

### Ultrasound Examinations

**Figure 1:**
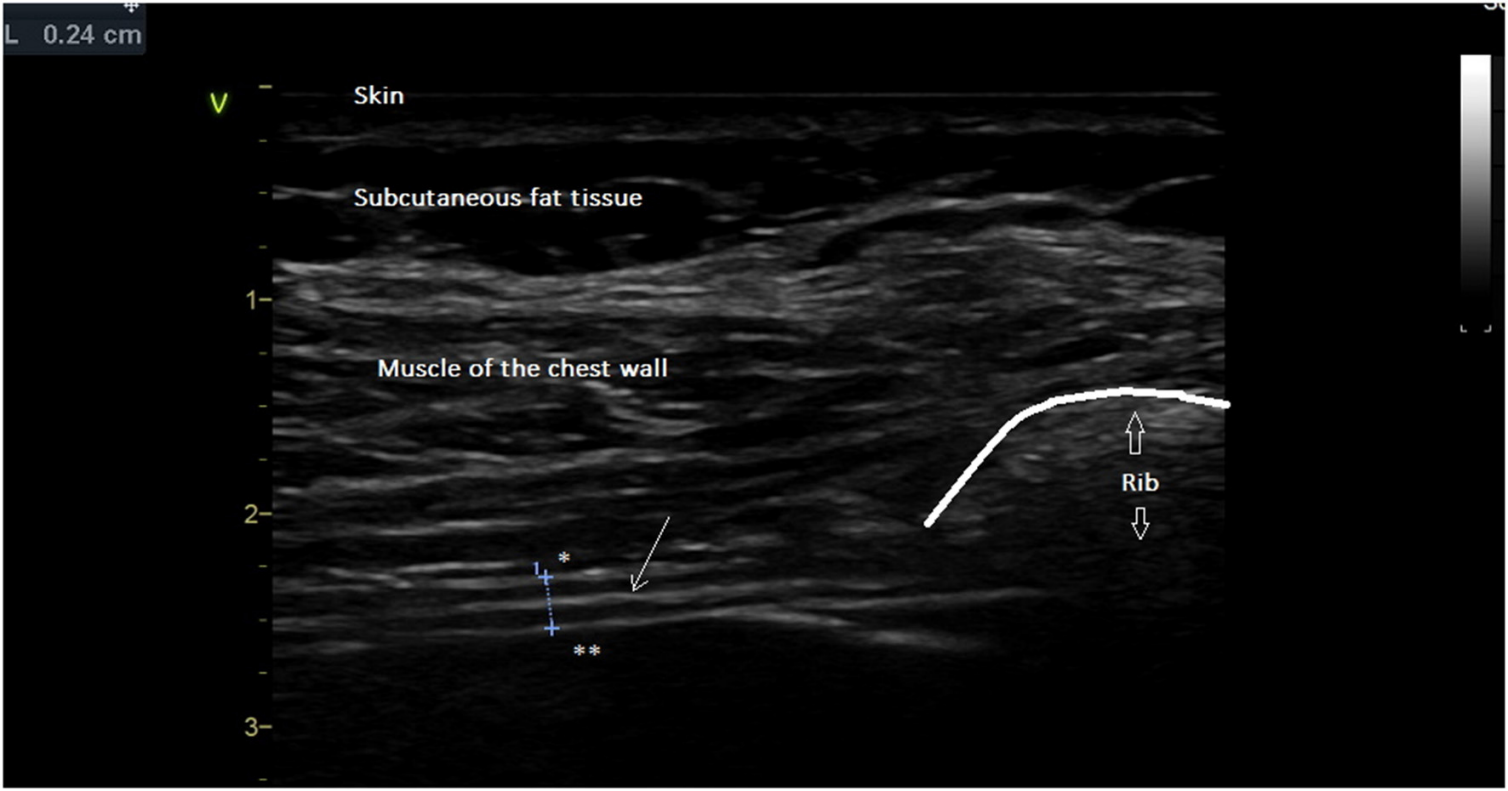
Diaphragm thickness at end-expiration (*L* = 0.24 cm) was measured from the middle of the pleural line * to the middle of the peritoneal line **. Thin arrow = fibrous center line. Large arrow = acoustic shadow generated by rib.

**Figure 2:**
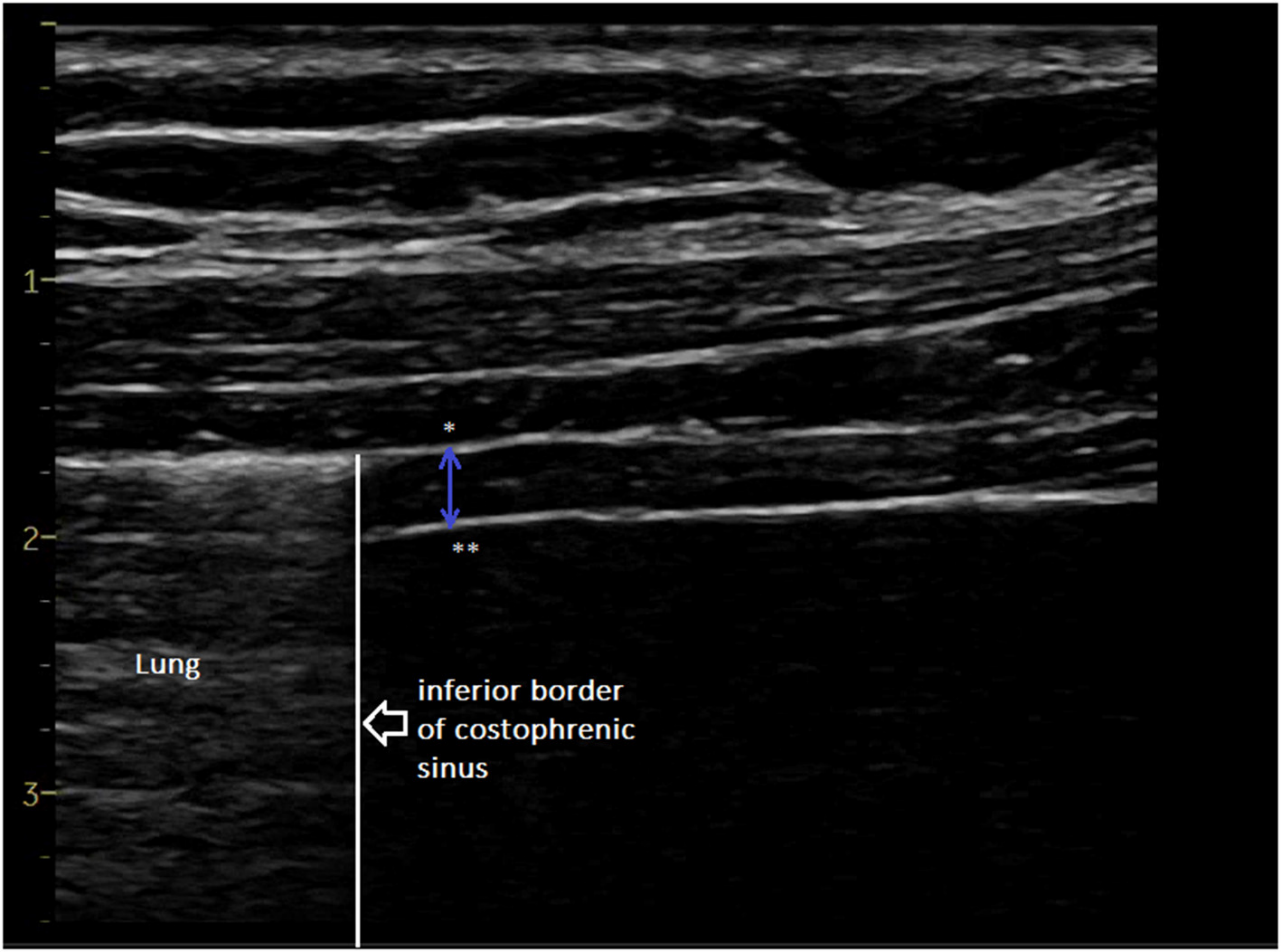
Diaphragm thickness at end-inspiration measured from the middle of the pleural line * to the middle of the peritoneal line **. Additional file: video reporting the changes in diaphragm thickness during the breathing cycle (increase in diaphragm thickness at inspiration).

The ultrasound examinations were carried out by two experienced investigators using a commercially available Doppler echocardiograph (Vivid S60N, GE Healthcare, Milwaukee, Wl, USA) connected to a linear vascular transducer (9L probe). The volunteers were studied on a chair, head and trunk aligned vertically and with hips and knees flexed at 90°. The trunk angle, i.e., the angle between the trunk and the horizontal axis through the trochanter, was around 90°. The diaphragmatic thicknesses were assessed by B-mode ultrasonography according to a previously published method (6, 7). Briefly, both hemidiaphragms were visualized at the zone of apposition, and the probe was placed below the phrenico-costal sinus near the anterior or the mid-axillary line at the eighth or the ninth intercostal space. The diaphragm was identified as a three-layered structure with two parallel echogenic lines, the diaphragmatic pleura and the peritoneal fascia, enclosing the hypoechoic diaphragmatic muscle. A third hyperechoic line was frequently seen in the middle of the non-echogenic layer, considered to be the fibrous layer in the center of the diaphragm. The intercostal space that provided the best visualization of the diaphragm was chosen and the probe was positioned as the two facial lines outlining the diaphragm were parallel. The thickness of each hemidiaphragm was directly measured from the frozen B-mode images (Figure 1). The diaphragm thickness was measured as the distance from the middle of the pleural membrane to the middle of the peritoneal membrane (14, 20, 21). The measurements were performed at the end of expiration (functional residual capacity), at the end of inspiration during quiet breathing at tidal volume (Figure 2), and after deep breathing at total lung capacity (TLC). Measurements were averaged from at least three different breathing cycles. All of the examinations were recorded for subsequent blind analysis. The percentage of thickening i.e., the ratio: thickness at end-inspiration–thickness at end-expiration divided by thickness at end-expiration, was determined for both hemidiaphragms for quiet breathing (at tidal volume) and maximal inspiration (at TLC). The ratio between the diaphragm thickness at the end of tidal volume and the diaphragm thickness at maximal inspiration (ΔTmax) was also calculated.

### Statistical Analysis

The results are reported as mean ± SD [lower limit of normal (LLN)–upper limit of normal (ULN)]. The lower and the upper limits of normal were calculated as means ± 1.95 SD. A linear regression analysis was carried out to probe for an association of the ultrasonographic measurements with gender, age, and body mass index. Statistical tests were performed with R statistical software. The significance level was *p* < 0.05.

## Results

To begin with, 216 healthy subjects were screened. However, 16 subjects could not be included because of lower spirometry parameters than normal, a poor echographic image quality, or a prior thoracic trauma. Two hundred healthy volunteers (100 men and 100 women) were ultimately included in the study. The characteristics of the healthy volunteers who were assessed are presented in Table 1.

**Table 1:** Study population: anthropometric data, pulmonary function tests and excursions of both hemidiaphragms recorded by M-mode ultrasonography.

	**Men**	**Women**
	**Mean ± SD**	**Mean ± SD**
Age (years)	51 ± 16	52 ± 16
Height (cm)	177 ± 7	163 ± 7
Weight (kg)	79 ± 12	67 ± 13
BMI (kg/m^2^)	25 ± 5	25 ± 5
SVC (L), (percentage of predicted %)	4.6 ± 0.9 (102 ± 11)	3.5 ± 0.9 (106 ± 13)
FVC (L), (percentage of predicted %)	4.6 ± 0.9 (101 ± 11)	3.4 ± 1 (104 ± 14)
FEV (L), (percentage of predicted %)	3.7 ± 0.7 (101 ± 11)	2.7 ± 0.8 (100 ± 12)
FEV / FVC ratio (%)	80 ± 6	81 ± 6
Right excursion—quiet breathing (cm)	2 ± 0.5	1.9 ± 0.5
Right excursion—voluntary sniffing (cm)	2.7 ± 0.7	2.3 ± 0.7
Right excursion—deep breathing (cm)	6 ± 0.9	5 ± 0.9
Left excursion—quiet breathing (cm)	2.2 ± 0.6	1.9 ± 0.5
Left excursion—voluntary sniffing (cm)	2.8 ± 0.8	2.4 ± 0.6
Left excursion—deep breathing (cm)	6.2 ± 0.9	5 ± 0.7

*BMI, body mass index; SVC, slow vital capacity; FVC, forced vital capacity; FEV1, forced expiratory volume in 1 s*.

Tables 2, 3 list the results of the diaphragmatic thicknesses measured in men and women on the right and the left side, respectively.

**Table 2:** Study of the right hemidiaphragm.

	**Men**	**Women**	***p***
	**Mean ± SD [LLN–ULN]**		
Thickness at end-expiration (FRC) (mm)	2.1 ± 0.4 [1.3–3]	1.9 ± 0.4 [1.1–2.7]	<0.001
Thickness at end-inspiration (QB) (mm)	2.8 ± 0.6 [1.7–3.9]	2.5 ± 0.6 [1.3–3.7]	<0.001
Percentage of thickening (QB) (%)	32 ± 15 [2–62]	35 ± 16 [4–67]	NS
Thickness at deep breathing (TLC) (mm)	4.3 ± 0.8 [2.8–5.9]	3.9 ± 0.8 [2.4–5.4]	<0.001
Thickening ratio	2.1 ± 0.3 [1.4–2.7]	2.2 ± 0.4 [1.4–2.9]	NS
Thickening fraction (%)	106 ± 34 [40–173]	116 ± 40 [39–193]	NS
ΔTmax	0.65 ± 0.1 [0.46–0.84]	0.64 ± 0.11 [0.43–0.86]	NS

*FRC, functional residual capacity; QB, quiet breathing; TLC, total lung capacity; LLN, lower limit of normality; ULN, upper limit of normality; ΔTmax, ratio between diaphragm thickness at the end of tidal volume and diaphragm thickness at maximal inspiration*.

**Table 3:** Study of the left hemidiaphragm.

	**Men**	**Women**	***p***
	**Mean ± SD [LLN–ULN]**		
Thickness at end-expiration (FRC)(mm)	2 ± 0.4 [1.3–2.7]	1.7 ± 0.3 [1.1–2.4]	<0.001
Thickness at end-inspiration (QB)(mm)	2.6 ± 0.5 [1.7–3.5]	2.3 ± 0.5 [1.3–3.3]	<0.001
Percentage of thickening (QB)(%)	30 ± 14 [4–57]	33 ± 15 [3–62]	NS
Thickness at deep breathing (TLC) (mm)	4.2 ± 0.8 [2.6–5.8]	3.8 ± 0.8 [2.3–5.3]	<0.001
Thickening ratio	2.1 ± 0.4 [1.4–2.8]	2.2 ± 0.4 [1.5–2.9]	NS
Thickening fraction (%)	112 ± 37 [39–184]	121 ± 37 [48–193]	NS
ΔTmax	0.63 ± 0.1 [0.45–0.8]	0.61 ± 0.1 [0.43–0.79]	NS

*FRC, functional residual capacity; QB, quiet breathing; TLC, total lung capacity; LLN, lower limit of normality; ULN, upper limit of normality; ΔTmax, ratio between diaphragm thickness at the end of tidal volume and diaphragm thickness at maximal inspiration*.

As expected, greater thicknesses were recorded in men compared to women on both sides and at the various breathing times studied, i.e., at end-expiration, end-inspiration during quiet breathing, and deep inspiration.

The right-to-left ratio of the thickness measured at end-expiration was ~1 and it was similar for both genders (1.1 ± 0.2 [0.7–1.5] in men and 1.1 ± 0.3 [0.6–1.6] in women.

The differences in the thicknesses measured at end-expiration for both sides were small and not significantly different in men (0.3 ± 0.3 mm [0–0.9]) vs. women (0.3 ± 0.3 mm [0–1]) The upper limit of normality in the difference of the thickness between the two sides was determined to be 0.9 mm in men and 1 mm in women.

The percentage of thickening at quiet breathing was similar in men and women. In the same individual, the difference in the percentage of thickening between the two sides was determined to be 13 ± 11% [0–34] in men and 16 ± 13% [0–41] in women. In some of the volunteers, the same percentage of thickening was recorded on both sides but in other subjects, the difference could be as much as 54%.

The percentage of thickening at deep breathing was not significantly different between men and women. In men, it ranged from 60 to 260% on the left side and from 57 to 200% on the right side. In women, the percentage of thickening at deep breathing ranged from 58 to 264% on the left side and from 60 to 229% on the right side. In the same individual, the difference between the two sides in the percentage of thickening at deep breathing, was 29 ± 23% [0–75] in men and 35 ± 29 % [0–92] in women.

The mean ΔTmax of both sides was calculated to be 0.64 ± 0.07 in men and 0.63 ± 0.08 in women. The upper limit of normality was calculated to be 0.79 in men and 0.78 in women. A number of healthy volunteers (20 women and 20 men) had a ΔTmax greater than 0.75 on one hemidiaphragm (right or left). By contrast, only one volunteer (a woman) out of 200 had a ΔTmax greater than 0.75 on both sides.

The results of the linear regression analysis correlation are presented in Table 4.

**Table 4:** Linear regression analysis assessing the association between the diaphragmatic thickness and the demographic and the BMI data.

	**Thickness (FRC)**		**Thickness (TLC)**		**Thickening fraction**	
	**Left**	**Right**	**Left**	**Right**	**Left**	**Right**
Gender (male)	<0.001	<0.001	<0.001	<0.0005	NS	NS
Age	NS	NS	NS	NS	NS	NS
BMI	<0.05	NS	NS	NS	NS	NS

*FRC, functional residual capacity; TLC, total lung capacity; BMI, Body Mass Index*.

## Discussion

This study determined the normal values of diaphragmatic thickness and the indices of contractility based on a large population of men and women assessed while in a seated position.

As suggested by previous studies (6, 7, 14) women had a thickness at end-expiration that was thinner than that of men. This indicates that gender-specific normal values should be used.

In our study, the lower limits of normality of the diaphragm thickness measured at end-expiration were estimated to be 1.1 mm in women and 1.3 mm in men on both sides. These values are close to the results of Cardenas et al. (14). Based on assessment of 64 healthy volunteers, these authors reported a lower limit of normality of the right hemidiaphragm of 1.23 mm in women and 1.25 mm in men. These thresholds should be useful for assessment of the quality of the diaphragm muscle and to detect atrophy induced by muscle wasting, neuromuscular disease, or hemidiaphragm paralysis (1, 22–25).

To estimate the quality of the diaphragm function it can be also instructive to compare the thickness of both sides measured at end-expiration by means of the right-to-left ratio. Boon et al. (6) have reported that one side is frequently thicker than the other in healthy volunteers, but that the difference between the two sides should be small in normal subjects. In our population, the difference between the two sides was small in men and women (a mean of 0.3 mm). The ratio of the thickness of both sides in patients with normal diaphragmatic function should be between 0.7 and 1.5 in men and between 0.6 and 1.6 in women. Furthermore, the upper limit of normality for the differences between sides was determined to be 1 mm in women and 0.9 mm in men. These thresholds can be used to detect a degree of imbalance between the two hemidiaphragms.

It has been shown that the use of indexes such as inspiratory thickening can be used to detect diaphragm dysfunction. In our work, the mean percentage of thickening during quiet breathing was determined to be between 30 and 35% on both sides in men and women, which is close to the results of Thimmaiah et al. (26).

As previously reported (27), a considerable degree of variability of thickening between the two sides has been noted. In our population, the mean difference in the percentage of thickening between the two sides was determined to be 13% in men and 16% in women. Although in some of the volunteers the same percentage of thickening was noted on both sides, in one woman the difference was nearly 54%. The upper limit of normality for the percentage of thickening at quiet breathing was determined to be between 57 and 67% according to the side and the gender. This value should be useful to detect an increase in the work of breathing at baseline. For example, this increase can be observed on one side in patients suffering from contralateral hemidiaphragm dysfunction (28). In such circumstances, the compensatory mechanism includes an increase in neural drive to the functioning hemidiaphragm (29) leading to an increase in muscular activity. The high degree of variability in the percentage of thickening during quiet breathing is the reason why in our population the lower limits of normality for the percentage of thickening during quiet breathing were close to 0 for men and women on both sides. The relevance of the assessment of the percentage of thickening during quiet breathing to assess diaphragmatic function has been questioned. Indeed, based on assessment of 150 healthy volunteers, Harper et al. (27) reported that in some healthy subjects (29% of the hemidiaphragms investigated) the percentage of thickening was less than 10% during tidal breathing. In three volunteers, a lack of thickening was observed on one side. In our population, the percentage of thickening at quiet breathing was less than 10% in five cases. Consequently, it is not possible to use the measurement of the thickening during quiet breathing to diagnose hemidiaphragm paralysis.

The use of the percentage of thickening at deep inspiration is recognized to be a better tool to detect hemidiaphragm paralysis. In our work, the thickening fraction was determined to range from 2.1 to 2.2 according to the side and gender. These results are in agreement with previous studies (9, 11, 14). Furthermore, the thickening fraction was ~100% in men and women, which is close to what was found in the study of Brown et al. (13) involving 45 healthy volunteers who were assessed while in a seated position. In contrast, these authors reported a lower mean thickening fraction in the same population when they were assessed while in a supine position (~60%).

To detect diaphragm dysfunction in the absence of complete paralysis, the lower limits of normality were assessed in our population. For the thickening ratio, the lower limit of normality was 1.4 in men on both sides. In women, the lower limit of normality was 1.4 on the right side and 1.5 on the left side.

For the thickening fraction, the thresholds were 35–38% in men and 35–47% in women for the right and the left hemidiaphragm, respectively. The lower limits of normality were calculated from the mean ± 1.95 SD, as recommended. Since there was a high degree of variability in our study in the thickening fraction between individuals, the thresholds may have been underestimated. In the whole population, no thickening fraction of less than 57% was observed. Consequently, a percentage of thickening greater than the LLN but lower than 57% should be considered as to be abnormally low.

In our results, the large range in the percentage of thickening suggested that various individual characteristics and probably technical aspects, such as the quality of the respiratory maneuver, the ultrasound image quality and the position of the probe, had an impact on the measurement of this parameter. In this context, it is not surprising that previous works have reported a poor correlation between the strength generated by the diaphragm, estimated by the transdiaphragmatic pressure, and the percentage of thickening (30). Further works would be interesting on this topic.

The ratio between the inspiratory thickness at quiet inspiration and the inspiratory thickness at deep breathing (ΔTmax) provides informations regarding the work of breathing at rest compared to the maximal work of breathing. A low ratio indicates a good capacity for the subject to increase the work of breathing (17). In contrast, a ΔTmax close to one suggests that the reserve of the increase in the work of breathing is minimal. In our work, the mean ΔTmax was determined to be 0.6. Fantini et al. (17) have reported that a ΔTmax greater than 0.75, is associated with an impairment in pulmonary function testing in ALS patients and an indication for mechanical ventilation support. When the ΔTmax is greater than 0.75, it provides 75% sensitivity and 85% specificity for predicting an FVC value lower than 50% of the predicted value. In our population, a ΔTmax greater than 0.75 at rest on one side could be observed in a number of the volunteers (40 out of 200 subjects). In patients, with an increase in the work of breathing at rest, the two hemidiaphragms should be stimulated. Consequently, for accurate detection of the limitation of the increase in the work of breathing, it would be better to measure the ΔTmax on both sides. In our population, a ΔTmax greater than 0.75 on both sides was observed in just one out of 200 volunteers. Furthermore, the determination of the mean ΔTmax of both sides should be informative. The upper limit for a normal mean ΔTmax was determined to be 0.78 in men and 0.79 in women.

The values determined in our work should only be used in patients assessed with the same procedure, i.e., in patients while seated and using the same method of measurements.

It has been reported that the hemidiaphragm thickness varies depending on which intercostal space is chosen, with hemidiaphragms being thicker at the lower intercostal space (6). In our work, the measurements performed on the intercostal space i.e., the 8^th^ or the 9^th^, provided the best image quality. It is not certain that the diaphragm thickness was systematically measured at the lower intercostal space, with an impact on the normal values proposed for the thickness at end-expiration and inspiration. Consequently, in clinical practice, when the measurements indicate a thin hemidiaphragm, it would be interesting to try a lower approach in an effort to confirm the limited thickness of the hemidiaphragm.

Furthermore, in this work, we chose to perform the measurements on BD images. There is no standardized approach for the measurement of diaphragm thickness. Some authors recommended the use of B-mode (6, 11) whereas other authors have used M-mode (15). It was recently reported that there is a good degree of agreement between both methods (31). Based on our experience in cardiac and vascular investigations, we believe that B-mode is preferable to M-mode. Indeed, to obtain a reliable measurement of the thickness it is important that the calipers are placed perpendicular to the hemidiaphragms. During breathing, cyclical motion of the diaphragm and the resulting changes in M-mode cursor location can result in a loss of perpendicularity of the M-line on the hemidiaphragm and hence a degree of error in the thickness measurement.

The limit values of normality of the thickness and the indexes of function of both hemidiaphragms reported in the present study should be useful to detect diaphragmatic dysfunction in the absence of complete paralysis. To the best of our knowledge, these thresholds are the first to have been determined with a large population of healthy volunteers of both genders while in a seated position. These findings are likely to be useful for guiding therapeutic management such as respiratory physiotherapy or the initiation of ventilatory support.

## Data Availability Statement

The raw data supporting the conclusions of this article will be made available by the authors, without undue reservation.

## Ethics Statement

The studies involving human participants were reviewed and approved by Aix Marseille University, CPPRB 1, NoA01299-32. The patients/participants provided their written informed consent to participate in this study.

## Author Contributions

AB and FB conceived and designed the study. SR, NV, J-JR, and JF assisted with the technical aspects of the protocol, recruited all the participants, and were involved in the acquisition of the data. AB and SR performed the ultrasound examinations. AB and GC analyzed the data and performed the statistical analysis. AB, GC, and SR have drafted the article while FB, J-JR, and NV revised it critically for important intellectual content. All authors have given final approval of the version to be published.

## Funding

This study was supported by a French Ministry of Defense research grant (Direction Générale de l'Armement, MHR-1-0723).

## Conflict of Interest

GC was employed by ALTRA BIO SA. The remaining authors declare that the research was conducted in the absence of any commercial or financial relationships that could be construed as a potential conflict of interest.

## Publisher's Note

All claims expressed in this article are solely those of the authors and do not necessarily represent those of their affiliated organizations, or those of the publisher, the editors and the reviewers. Any product that may be evaluated in this article, or claim that may be made by its manufacturer, is not guaranteed or endorsed by the publisher.
